# Genotype by Selenium Concentration Interactions for Selenium-Related Genes in *Paramisgurnus dabryanus* at Different Durations

**DOI:** 10.3390/ani16182837

**Published:** 2026-09-09

**Authors:** Zhihui Huang, Xin’an Wang, Benhe Ma, Ruiyu Deng, Yunyi Gao, Guohong Yu, Chuanduan Ke, Haihua Wang, Aijun Ma

**Affiliations:** 1 State Key Laboratory of Mariculture Biobreeding and Sustainable Goods, Yellow Sea Fisheries Research Institute, Chinese Academy of Fishery Sciences, China-ASEAN Belt and Road Joint Laboratory on Mariculture Technology (Qingdao), Qingdao 266071, China; 2 Jiangxi Fisheries Research Institute, Nanchang 330039, China; 3 Jiujiang Xingxiang Aquatic Breeding Co., Ltd., Jiujiang 332200, China

**Keywords:** *P. dabryanus*, selenium-related genes, selenium concentration, gene-by-environment interaction

## Abstract

To distinguish the direct regulatory effect of nano-selenium on selenoprotein pathways from its indirect induction of glutathione metabolism, we measured the expression of eight genes representing these two core pathways (*selenoo1*, *trnau1apl*, *selenop*, and *gpx1* for selenoprotein synthesis; *mgst1*, *cha2*, *hagh*, and *glo1* for glutathione metabolism) in *Paramisgurnus dabryanus* at 20, 40, and 60 days of feeding, enabling the characterisation of short-, medium- and long-term transcriptional responses. Additive main effects and multiplicative interaction (AMMI) and genotype environment interaction (GGE) biplots were used to analyse the genetic basis of expression of the two types of selenium-related genes. The AMMI analysis of the expression of two types of selenium-related genes in *P. dabryanus* showed that, for selenoprotein-synthesis-related genes, the effect of selenium concentration decreased slightly and then gradually increased over time. For glutathione metabolism-related genes, the effect of selenium concentration decreased linearly over time. For both gene types, as time progressed, a clear antagonistic relationship was observed between the genotype effect and the genotype × selenium concentration interaction. GGE biplot analysis indicated that, overall, for selenoprotein-synthesis-related genes, the top two based on comprehensive evaluation of both stability and high expression were *gpx1* and *selenop*. *gpx1* may serve as a potential candidate for assessing nano-selenium bioavailability, selenium nutritional status, and antioxidant responses, warranting further validation. Overall, the top two glutathione metabolism-related genes in terms of comprehensive evaluation were *glo1* and *hagh*. *glo1* may be a potential indicator of nano-selenium-induced oxidative stress or glutathione metabolic load.

## Simple Summary

Selenium is an essential trace mineral for fish, but its effects depend on both the amount given and the duration of feeding. This study examined how different dietary selenium levels and feeding times affect the activity of eight selenium-related genes in the foregut of the large-scale loach, an economically important freshwater fish in China. We used two statistical methods, AMMI and GGE biplot, to analyse the interaction between genes and added nano-selenium concentrations at 20, 40 and 60 days of feeding. For selenoprotein-synthesis genes (*selenoo1*, *trnau1apl*, *selenop* and *gpx1*), the influence of selenium concentration first slightly decreased and then increased over time. For glutathione metabolism genes (*mgst1*, *cha2*, *hagh* and *glo1*), the selenium effect steadily decreased over time. Among selenoprotein genes, *gpx1* stood out as having the highest expression and good stability, making it a potential biomarker for assessing selenium status and antioxidant responses. Among glutathione genes, *glo1* showed the strongest and most stable expression, suggesting it may serve as a potential indicator of selenium-induced oxidative stress or glutathione metabolic load. These findings help clarify how different genes respond to selenium over time and provide a molecular basis for optimising selenium supplementation strategies in loach farming.

## 1. Introduction

Selenium (Se), as an essential trace element for aquatic animals, plays irreplaceable physiological roles in maintaining body health, regulating antioxidant defence systems and promoting growth performance. The molecular basis of these functions lies in its incorporation into selenoproteins (e.g., glutathione peroxidase, GSH-Px), which scavenge harmful peroxides and protect biological membrane structures from oxidative damage [1,2]. However, the biological effects of selenium exhibit a typical “U-shaped” dose–response relationship, where both deficiency and excess lead to metabolic disorders, immune suppression, and even toxic manifestations [3,4]. This characteristic necessitates precise regulation of selenium supplementation in aquafeeds. Traditional inorganic selenium sources (e.g., sodium selenite) suffer from low bioavailability and high toxicity risk [5,6], whereas organic selenium (e.g., seleno-yeast) exhibits higher bioavailability but does not fully overcome the narrow safety window [5]. In recent years, nano-selenium has emerged as a research hotspot in aquatic nutrition due to its unique surface effects and small particle size, exhibiting higher biological activity and improved safety [6,7]. Specifically, biosynthetically prepared nano-selenium has been shown to optimise gut microbiota composition, activate antioxidant enzyme systems, and upregulate immune-related gene expression, thereby significantly improving the health status of aquatic animals [8]. However, the mechanisms underlying the metabolic response of different aquatic species to nano-selenium remain to be systematically elucidated [9].

*Paramisgurnus dabryanus*, belonging to the order Cypriniformes, family Cobitidae, and subfamily Cobitinae, is a benthic fish characterised by high protein and low fat content [10]. It is an important economic freshwater fish species endemic to China. Its meat is tender and nutritious, with high crude protein and low fat, and it exhibits strong environmental adaptability. Its breeding technology is becoming increasingly mature, and it has become a leading species in the aquaculture industry. With the growing intensity of intensive farming, enhancing disease resistance, improving meat quality, and achieving healthy farming through nutritional regulation have become practical problems facing the industry. Selenium, as a key functional trace element, is increasingly recognised for its role in the healthy aquaculture system of loach.

In recent years, significant progress has been made in the study of selenium nutrition for *P. dabryanus*. However, existing research has mostly focused on phenotypic indicators such as growth and antioxidant activity, while the exploration of underlying mechanisms—for example, how nano-selenium affects the transcriptional expression of selenium-metabolism-related genes—remains superficial. In particular, a critical knowledge gap persists regarding genotype-by-selenium-concentration interactions. Different genetic backgrounds may respond divergently to dietary selenium levels, as gene expression is governed by both intrinsic genetic factors and extrinsic nutritional stimuli. Yet, most previous studies in fish selenium nutrition have treated genes as independent responders to selenium supplementation, rather than examining how genetic differences modulate the magnitude or direction of selenium-induced transcriptional changes. This omission is significant because ignoring genotype-by-environment interactions may lead to oversimplified recommendations for selenium supplementation, and may obscure genes that are stably expressed across conditions versus those that are responsive to selenium dose. As a result, the relative contribution of genotypic effects versus genotype-by-selenium-concentration interactions remains unexplored in *P. dabryanus* nutrition. Without this knowledge, it is difficult to predict whether a gene identified as a biomarker under one selenium regime will remain reliable under different dietary conditions, or whether selenium supplementation strategies need to be tailored to specific genetic backgrounds in loach farming. To address this gap, we employed the additive main effects and multiplicative interaction (AMMI) model [11] and the genotype main effects plus genotype × environment interaction (GGE) biplot analysis [12]. In these models, each gene is treated as a “genotype” and each selenium concentration as an “environment”, allowing the decomposition of expression variation into gene-specific, concentration-specific, and interactive components. Unlike ANOVA, which only provides F-statistics, AMMI quantifies the relative contribution of each factor to total variation (as % of total SS), while GGE biplot enables visualisation of gene rankings across concentrations and identification of genes with high and stable expression—information that is critical for biomarker screening but cannot be obtained from univariate approaches. These methods have been widely used in plant breeding [11,12] but have seldom been applied to fish selenium nutrition.

In the present study, we therefore used AMMI and GGE biplot to analyse the effect of genotype × selenium concentration interaction on the expression of two functional categories of selenium-related genes in the foregut of *P. dabryanus.* at 20, 40 and 60 days of feeding. The first category includes selenoprotein synthesis-related genes: *selenoo1* (involved in endoplasmic reticulum redox regulation and protein folding) [13], *trnau1apl* (also known as SECp43, a selenocysteine-specific tRNA-associated protein required for selenoprotein translation, which enhances the efficiency of selenoprotein synthesis by mediating interactions between SECIS binding protein 2 and the Sec-tRNA complex) [14], *selenop* (the main selenium transporter in plasma, responsible for distributing selenium from the liver to peripheral tissues) [15,16], and *gpx1* (encodes glutathione peroxidase 1, a key antioxidant enzyme that protects cells from oxidative damage by reducing hydrogen peroxide and organic hydroperoxides) [17]. The second category comprises glutathione metabolism-related genes: *mgst1* (microsomal glutathione S-transferase 1, involved in the detoxification of xenobiotics and oxidative stress products) [18], *cha2* (also known as GCLM, the regulatory subunit of glutamate-cysteine ligase, which modulates the rate-limiting enzyme in glutathione biosynthesis) [19], *hagh* (hydroxyacyl glutathione hydrolase, also known as glyoxalase II, responsible for the hydrolysis of S-lactoyl-glutathione to generate reduced glutathione and D-lactate) [20], and *glo1* (glyoxalase 1, which catalyses the detoxification of methylglyoxal, a toxic by-product of glycolysis, via condensation with glutathione) [21]. These genes were selected because they represent the core pathways of selenium utilisation (selenoprotein synthesis) and the non-selenium-dependent antioxidant/glutathione system [22]. By comparing their expression patterns across selenium concentrations and time, we aimed to distinguish the direct regulatory effect of nano-selenium on selenoprotein pathways from its indirect induction of glutathione metabolism, thereby revealing potential synergistic or compensatory relationships in maintaining selenium homeostasis. The results provide a molecular basis for optimising nano-selenium supplementation strategies in loach aquaculture. We hypothesised that: (1) genotype × selenium concentration interaction significantly affects the expression of the eight selenium-related genes; (2) selenoprotein-synthesis-related and glutathione-metabolism-related genes exhibit different temporal response patterns to selenium concentration; and (3) genes with high and stable expression across concentrations can be identified as potential candidates for further biomarker development. Although this study does not directly model human biology, it contributes to understanding selenium metabolism in vertebrates and may inform future comparative studies.

## 2. Materials and Methods

### 2.1. Ethics Statement

All experimental treatments for artificially cultivated fish were performed according to the recommendations in the Guide for the Care and Use of Laboratory Animals of the National Institutes of Health, China. The study protocol followed the recommendations of the Experimental Animal Ethics Committee, Yellow Sea Fisheries Research Institute, Chinese Academy of Fishery Sciences, China (Decision No: YSFRI-2025079).

### 2.2. Experimental Materials

In this experiment, juvenile *P. dabryanus* were fed five diets with different selenium levels, all within an appropriate range. The composition and nutritional components of the basic feed formula are shown in Table 1. Nano-selenium from Yunnan Boshiao Biotechnology Co., Ltd., (Kunming, China) was used as the selenium source. It was added to the basal diet at five levels: 0, 0.5 ± 0.01, 1.0 ± 0.01, 2.0 ± 0.02, and 3.0 ± 0.015 mg/kg, corresponding to addition rates of 0%, 0.01%, 0.02%, 0.04%, and 0.06% of the diet, respectively. These groups were designated as A, B, C, D, and E. Each feed ingredient is crushed and passed through a 40-mesh sieve. Pure water, amounting to 15% of the weight of each ingredient, is added and mixed. The mixture was then made into sinking pellet feed using a pellet mill at room temperature (not exceeding 40 °C during processing) and air-dried to minimise potential degradation of nano-selenium. The final selenium content of each diet was measured by inductively coupled plasma mass spectrometry (ICP-MS) at Qingdao Standard Testing Co., Ltd. (Qingdao, China) (Table 1).

### 2.3. Experimental Methods

#### 2.3.1. Experimental Fish and Experimental Design

The *P. dabryanus* juveniles were obtained from a commercial hatchery and were clinically healthy upon arrival. The fish were temporarily raised for 20 days in the aquaculture circulation system of Jiangxi Fisheries Research Institute, (Nanchang, China). With the water temperature maintained at 27 ± 0.5 °C, continuous aeration ensures a dissolved oxygen level of ≥7.5 mg/L. Feed commercial bait once a day in the morning and once in the evening. Prior to the formal experiment, 1050 healthy and uniform loach juveniles were selected, fasted for 24 h, and their initial growth indicators were measured, revealing an initial body weight of (14.15 ± 1.22) g. Sex was not determined because juvenile fish lack external sexual dimorphism. The fish were then randomly allocated to fibreglass aquaculture tanks (300 L water volume) with three replicates per group and 70 fish per replicate. The experiment lasted for 60 days. During the experiment, the water temperature was maintained at 27 ± 0.5 °C, the pH was 7.5 ± 0.5, and continuous aeration and oxygenation were provided 24 h a day, DO ≥ 7.5 ± 0.5 mg/L. Throughout the experiment, feeding rations were initially set at 3–5% of body weight and then adjusted weekly in response to the fish’s feeding behaviour. Weekly sampling and testing of water quality parameters (e.g., temperature, dissolved oxygen, pH) were conducted to maintain a stable breeding environment. Following a feeding period, subject the *P. dabryanus* to a 24 h fast. Then, measure their growth indicators. The loaches were fed with different nano-selenium diets. All fish that remained healthy throughout the trial were eligible for sampling, and no data points were excluded. Blinding was not applied in this study because the same investigator performed all experimental procedures, sample collection, and data analysis. Fish were euthanized by anaesthetic overdose at the end of the experiment.

#### 2.3.2. Sample Collection

During the breeding experiment, samples were collected on days 20, 40, and 60. Food was withheld 24 h prior to sampling, and the fish were anaesthetised using eugenol (0.02%). The foregut was selected as the target tissue based on preliminary experiments, showing the most robust and reproducible expression of the target genes compared with other tissues (liver, muscle, and gill), consistent with its role as the primary site of nutrient absorption. Four fish were randomly selected from each breeding tank, and their foregut tissue was dissected on a chilled surface and immediately placed in RNA preservation solution. Thus, for each of the five selenium concentrations and each time point (20, 40, 60 d), a total of 12 fish (4 fish × 3 replicate tanks) were sampled for gene expression analysis, giving *n* = 12 per experimental group per time point. After overnight storage at 4 °C, the samples were transferred to a −80 °C freezer for subsequent mRNA expression analysis. At the 20 day and 40 day sampling points, fish were anaesthetised with eugenol for tissue collection and were allowed to recover after sampling; only at the end of the 60 day experiment were all remaining fish euthanized by anaesthetic overdose.

#### 2.3.3. Expression Analysis of Key Functional Genes Involved in Selenium-Rich Traits

Total RNA was extracted from tissue samples using a commercial kit (Tiangen Biotech (Beijing) Co., Ltd., Beijing, China). The RNA concentration and purity were determined with a Nanodrop 2000 spectrophotometer (Thermo Fisher Scientific, Waltham, MA, USA), and its integrity was assessed by agarose gel electrophoresis. cDNA was synthesised using the HiScript III RT SuperMix for qPCR (+gDNA wiper) assay kit (Vazyme Biotech Co., Ltd., Nanjing, China). Gene-specific primers for qPCR were designed using Premier 5.0 software. The sequences of the primers used are listed in Table 2. Primer efficiency for each gene was determined by standard curve analysis using a five-point serial dilution of pooled cDNA, with efficiencies ranging from 90% to 110% for all primer pairs. Melt-curve analysis was performed after each run to confirm amplification of single specific products, and only samples showing a single sharp peak were used for subsequent analysis. Detailed information on primer efficiency, slope, R^2^, and melt-curve analysis for each primer pair is provided in Supplementary Materials Table S1. qPCR was performed using the StepOne Plus Real-Time PCR System (Thermo Fisher Scientific, Waltham, MA, USA). The reaction was performed in a 20 μL system containing: 10 μL of 2× SYBR Green Pro Taq HS Premix (Hunan Aikerui Bioengineering Co., Ltd., Changsha, China), 0.8 μL each of the forward (F) and reverse (R) primers (10 μmol/L), 2 μL of cDNA, and 6.4 μL of DEPC water. The reaction procedure is: 50 °C, 2 min; 95 °C, 30 s; 95 °C, 40 cycles of 15 s; 58 °C, 45 s; 95 °C, 15 s; 72 °C, 5 min.

Based on our previous research, using *ef1α* and *β-actin* as an internal reference gene [23,24], relative expression levels were calculated according to Hellemans and Vandesompele (2014) [25]. Values are presented as mean ± SD. The experimental data were processed using Excel 2007 and SPSS 19.0 software, and one-way ANOVA was used for analysis. A *p*-value of less than 0.05 was considered statistically significant.

No a priori sample size calculation was performed. The sample size (70 fish per tank, 3 tanks per group) was chosen based on previous similar studies and the practical capacity of the aquaculture system [26].

### 2.4. Data Analysis

#### 2.4.1. AMMI Analysis

The main feature of the AMMI model is that it integrates analysis of variance and principal component analysis (PCA) [11]. The AMMI model for the *g*th genotype (*selenoo1*, *trnau1apl*, *selenop*, and *gpx1*/*mgst1*, *cha2*, *hagh*, and *glo1*) in the *e*th added nano-selenium concentration (0, 0.5, 1, 2, and 3 mg/kg) was as follows:

$$y_{ge}=\mu +\alpha_{g}+\beta_{e}+\sum_{i=1}^{N}\lambda_{n}\gamma_{gn}\delta_{en}+\theta_{ge}$$

where $y_{ge}$ is the expression of the four genotypes g in selenium concentration e; μ is the grand mean; *α_g_* is the average deviation of genotypes (the average value of each genotype minus the grand average value); *β_e_* is the average deviation of the selenium concentration (the average of each selenium concentration minus the grand average); *λ_n_* is the eigenvalue of the *n*th interaction principal component axis (IPCA); *γ_gn_* is the genotype principal component score of the nth principal component; *δ_en_* is the selenium concentration principal component score of the *n*th principal component; N is the total number of principal component axes; and *θ_ge_* is the residual.

#### 2.4.2. GGE Biplot Analysis

GGE biplot analysis can reveal the complex interaction between different factors [27]. The gene expression data obtained from different selenium concentration experiments were sorted into a two-way table, in which each value was the average value of the expression of the corresponding genes in the corresponding selenium concentration (i.e., the phenotype value (*y_ge_*)). Singular value decomposition of the first two principal components was used to fit the GGE biplot model [12] as follows:

$$y_{ge}=\mu +\beta_{e}+\lambda_{1}\gamma_{g1}\delta_{e1}+\lambda_{2}\gamma_{g2}\delta_{e2}+\theta_{ge}$$

where $y_{ge}$ is the trait mean expression for genotype *g* in selenium concentration *e*; *μ* is the grand mean; $\beta_{e}$ is the main effect of selenium concentration *e*; $\mu +\beta_{e}$ is the mean expression across all genotypes in selenium concentration *e*; $\lambda_{1}$ and $\lambda_{2}$ are the singular values for the first and second principal components (PC1 and PC2), respectively; $\gamma_{g1}$ and $\gamma_{g2}$ are eigenvectors of genotype *g* for PC1 and PC2, respectively; $\delta_{e1}$ and $\delta_{e2}$ are eigenvectors of selenium concentration *e* for PC1 and PC2, respectively; and $\theta_{ge}$ is the residual associated with genotype *g* in selenium concentration *e*.

The AMMI and GGE biplot were performed by DPS Data Processing System [28].

## 3. Results

### 3.1. Selenoprotein Synthesis-Related Genes (selenoo1, trnau1apl, selenop, and gpx1)

The raw mean expression values for each gene at each added nano-selenium concentrations and time point are provided in Supplementary Materials Table S2.

#### 3.1.1. AMMI Analysis of Selenoprotein Synthesis-Related Genes

The AMMI analysis revealed that the expression of the selenium-related genes *selenoo1*, *trnau1apl*, *selenop*, and *gpx1* was extremely significantly affected by genotype, selenium concentration, and genotype × selenium concentration interactions at three durations (Table 3). At 20 d, 33.94248%, 9.847928% and 46.79153% of the total SS (sum of squares) were attributable to the genotype, selenium concentration effect, and the genotype × selenium concentration interaction, respectively; at 40 d, the values were 74.56188%, 7.300298% and 12.96041%; and at 60 d, they were 30.8304%, 26.6049% and 32.6851%.

**Table 3:** AMMI analysis results for selenium-related genes *selenoo1*, *trnau1apl*, *selenop*, and *gpx1* in different selenium concentration at different duration.

Durations	Source of Variation	*df*	SS	MS	*F*	Prob.	% of Total SS
20 d	Total	79	50.108	0.6343			
	Treatment	19	45.3887	2.3889	30.3721	*p* < 0.0001	
	Gene	3	17.0079	5.6693	72.0792	*p* < 0.0001	33.94248
	Concentration	4	4.9346	1.2337	15.6846	*p* < 0.0001	9.847928
	Interaction	12	23.4463	1.9539	24.8412	*p* < 0.0001	46.79153
	IPCA1	6	21.83507	3.63918	46.2684	*p* < 0.0001	
	IPCA2	4	1.47071	0.36768	4.67463	0.002374	
	Residual	2	0.14047	0.07023			
	Error	60	4.71922	0.07865			
40 d	Total	79	39.9833	0.5061			
	Treatment	19	37.9131	1.9954	57.8349	*p* < 0.0001	
	Gene	3	29.8123	9.9374	288.0232	*p* < 0.0001	74.56188
	Concentration	4	2.9189	0.7297	21.1499	*p* < 0.0001	7.300298
	Interaction	12	5.182	0.4318	12.5162	*p* < 0.0001	12.96041
	IPCA1	6	3.76023	0.62671	18.16425	*p* < 0.0001	
	IPCA2	4	1.35356	0.33839	9.80778	*p* < 0.0001	
	Residual	2	0.06825	0.03412			
	Error	60	2.07013	0.0345			
60 d	Total	79	24.2196	0.3066			
	Treatment	19	21.8268	1.1488	28.8065	*p* < 0.0001	
	Gene	3	7.467	2.489	62.4136	*p* < 0.0001	30.8304
	Concentration	4	6.4436	1.6109	40.3948	*p* < 0.0001	26.6049
	Interaction	12	7.9162	0.6597	16.5419	*p* < 0.0001	32.6851
	IPCA1	6	7.1701	1.19502	29.96593	*p* < 0.0001	
	IPCA2	4	0.62493	0.15623	3.91764	0.006836	
	Residual	2	0.12112	0.06056			
	Error	60	2.39275	0.03988			

Note: SS: sum of squares; *df*: degrees of freedom; MS: mean squares; *p* < 0.05; *p* < 0.01; *p* < 0.0001 were considered significant, highly significant, and extremely significant, respectively. Same meaning as abbreviations in Table 4.

**Table 4:** AMMI analysis results for selenium-related genes *mgst1*, *cha2*, *hagh*, and *glo1* in different selenium concentrations at different durations.

Durations	Source of Variation	*df*	SS	MS	*F*	Prob.	% of Total SS
20 d	Total	79	76.822	0.9724			
	Treatment	19	72.4656	3.814	52.5289	*p* < 0.0001	
	Gene	3	40.1635	13.3878	184.387	*p* < 0.0001	52.28125
	Concentration	4	18.9761	4.744	65.3381	*p* < 0.0001	24.70139
	Interaction	12	13.326	1.1105	15.2945	*p* < 0.0001	17.34659
	IPCA1	6	8.88133	1.48022	20.38667	*p* < 0.0001	
	IPCA2	4	3.03803	0.75951	10.46049	*p* < 0.0001	
	Residual	2	1.40659	0.70329			
	Error	60	4.35644	0.07261			
40 d	Total	79	36.0267	0.456			
	Treatment	19	33.3148	1.7534	38.7944	*p* < 0.0001	
	Gene	3	10.9378	3.6459	80.6666	*p* < 0.0001	30.36026
	Concentration	4	7.5539	1.8885	41.7826	*p* < 0.0001	20.9675
	Interaction	12	14.8231	1.2353	27.3303	*p* < 0.001	41.14476
	IPCA1	6	12.00245	2.00041	44.25923	*p* < 0.0001	
	IPCA2	4	2.40194	0.60049	13.28581	*p* < 0.0001	
	Residual	2	0.41875	0.20937			
	Error	60	2.71185	0.0452			
60 d	Total	79	256.2192	3.2433			
	Treatment	19	245.6162	12.9272	73.1521	*p* < 0.0001	
	Gene	3	123.7225	41.2408	233.3729	*p* < 0.0001	48.28776
	Concentration	4	43.2032	10.8008	61.1194	*p* < 0.0001	16.86181
	Interaction	12	78.6905	6.5575	37.1077	*p* < 0.0001	30.71218
	IPCA1	6	67.33461	11.22244	63.50534	*p* < 0.0001	
	IPCA2	4	7.13524	1.78381	10.0942	*p* < 0.0001	
	Residual	2	4.22061	2.1103			
	Error	60	10.60298	0.17672			

#### 3.1.2. GGE Biplot Analysis of Selenoprotein Synthesis-Related Genes

**Figure 1:**
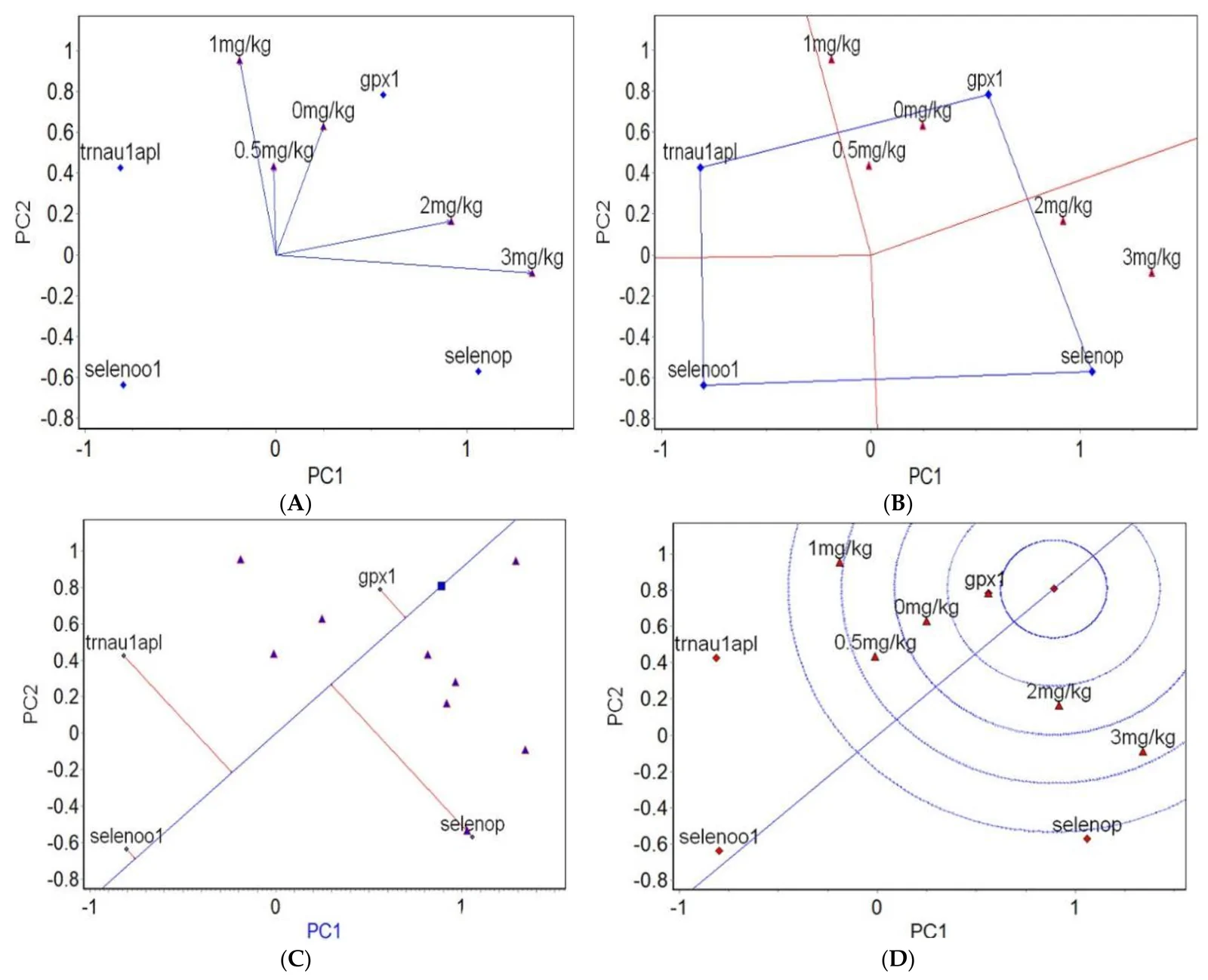
GGE biplot of selenium-related genes *selenoo1*, *trnau1apl*, *selenop*, and *gpx1* in different selenium concentrations at 20 d. PC1 and PC2 explained 57.01% and 31.88% of the total variation, respectively. For detailed explanations of each view, see Supplementary Materials Section S2. (**A**) The relationship among different concentration; (**B**) “Which-won-where” view; (**C**) “High expression and expression stability” view; (**D**) GGE biplot with concentric circles.

**Figure 2:**
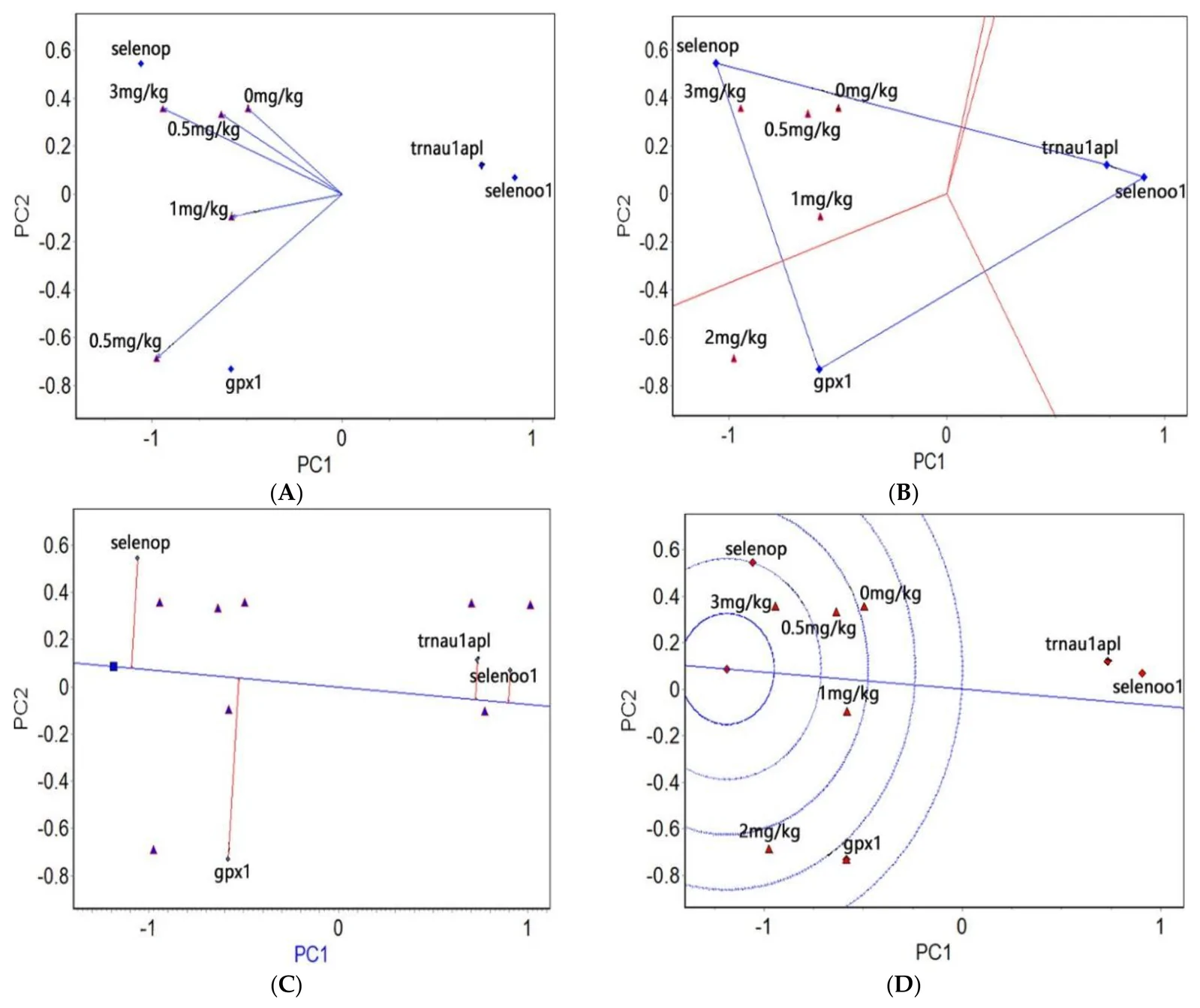
GGE biplot of selenium-related genes *selenoo1*, *trnau1apl*, *selenop*, and *gpx1* in different selenium concentrations at 40 d. PC1 and PC2 explained 72.8% and 21.9% of the total variation, respectively. For detailed explanations of each view, see Supplementary Materials Section S2. (**A**) The relationship among different concentrations; (**B**) “Which-won-where” view; (**C**) “High expression and expression stability” view; (**D**) GGE biplot with concentric circles.

**Figure 3:**
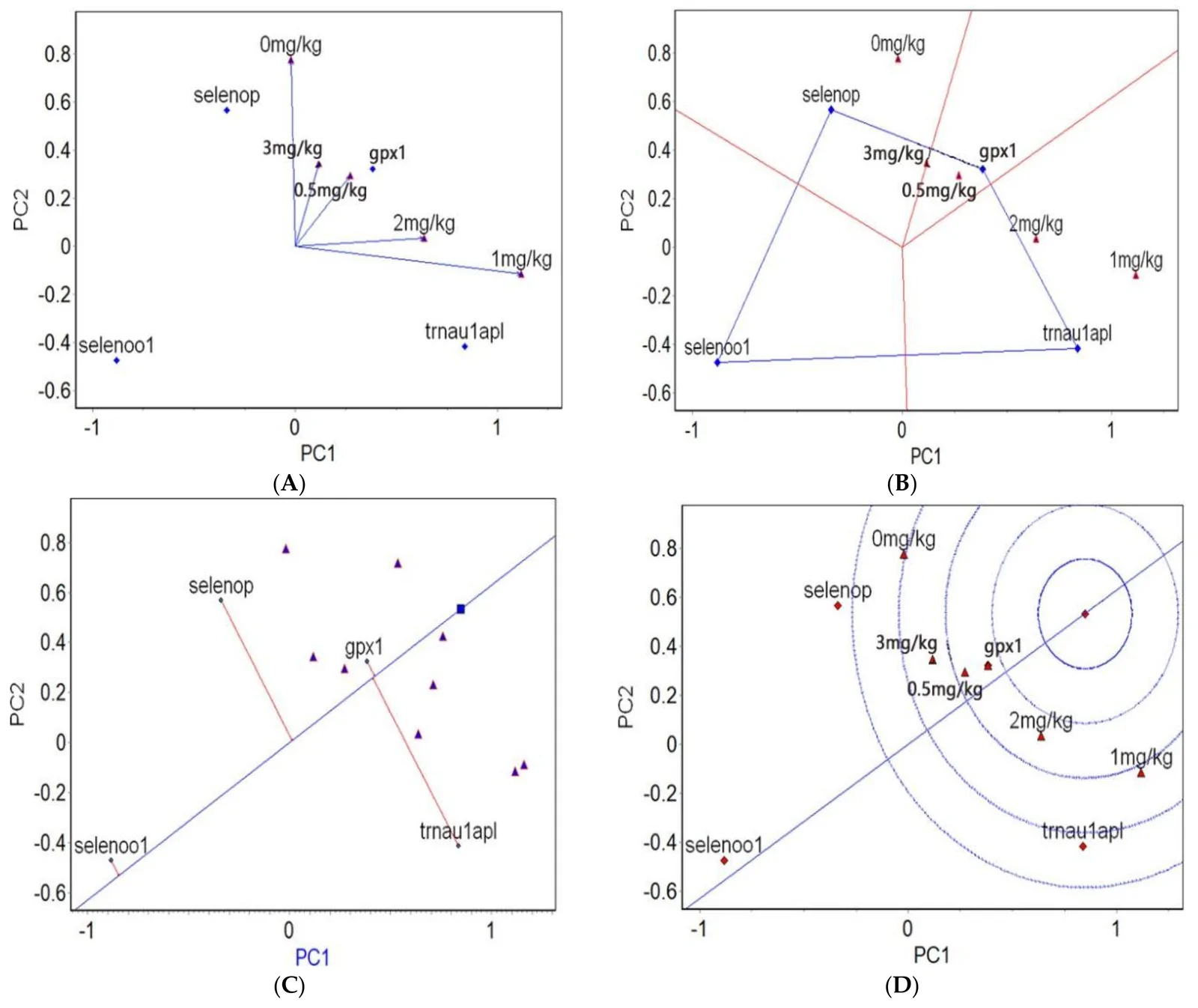
GGE biplot of selenium-related genes *selenoo1*, *trnau1apl*, *selenop*, and *gpx1* in different selenium concentrations at 60 d. PC1 and PC2 explained 59.42% and 28.05% of the total variation, respectively. For detailed explanations of each view, see Supplementary Materials Section S2. (**A**) The relationship among different concentrations; (**B**) “Which-won-where” view; (**C**) “High expression and expression stability” view; (**D**) GGE biplot with concentric circles.

**Figure 4:**
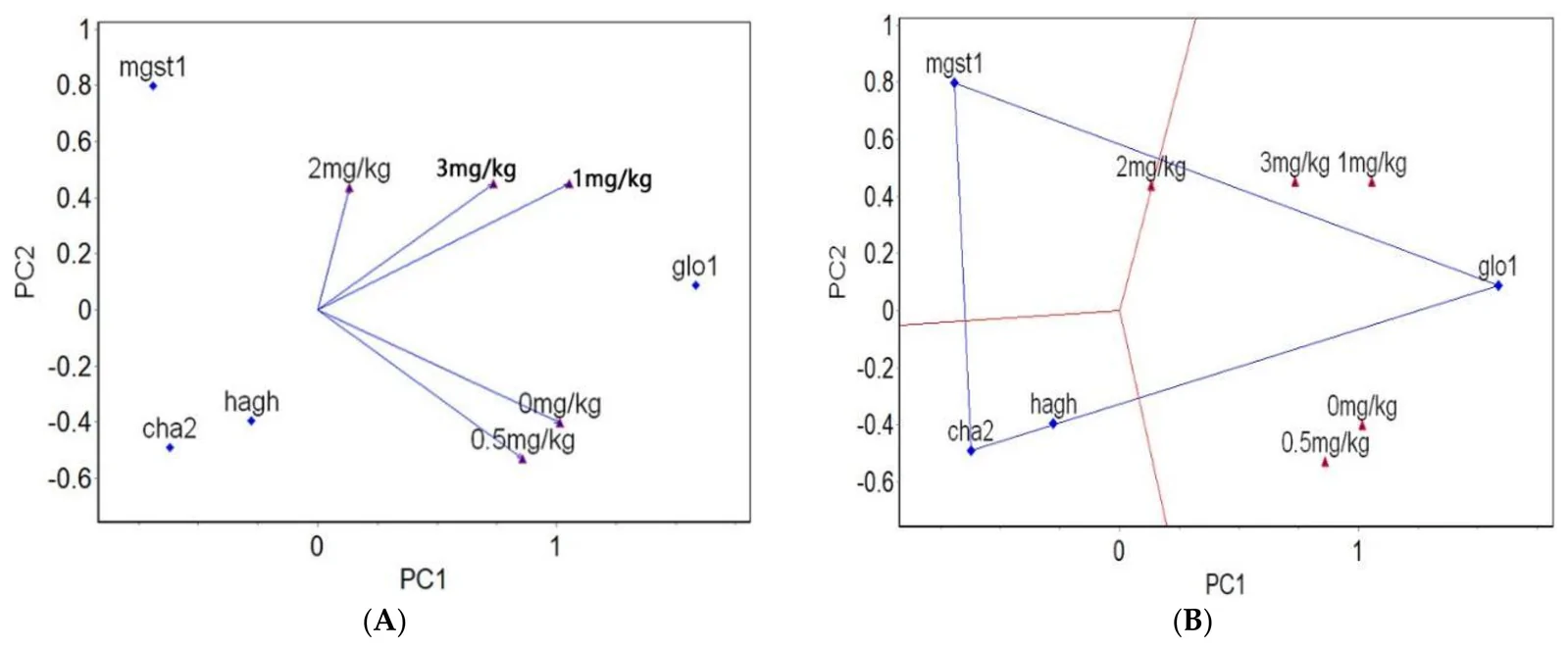

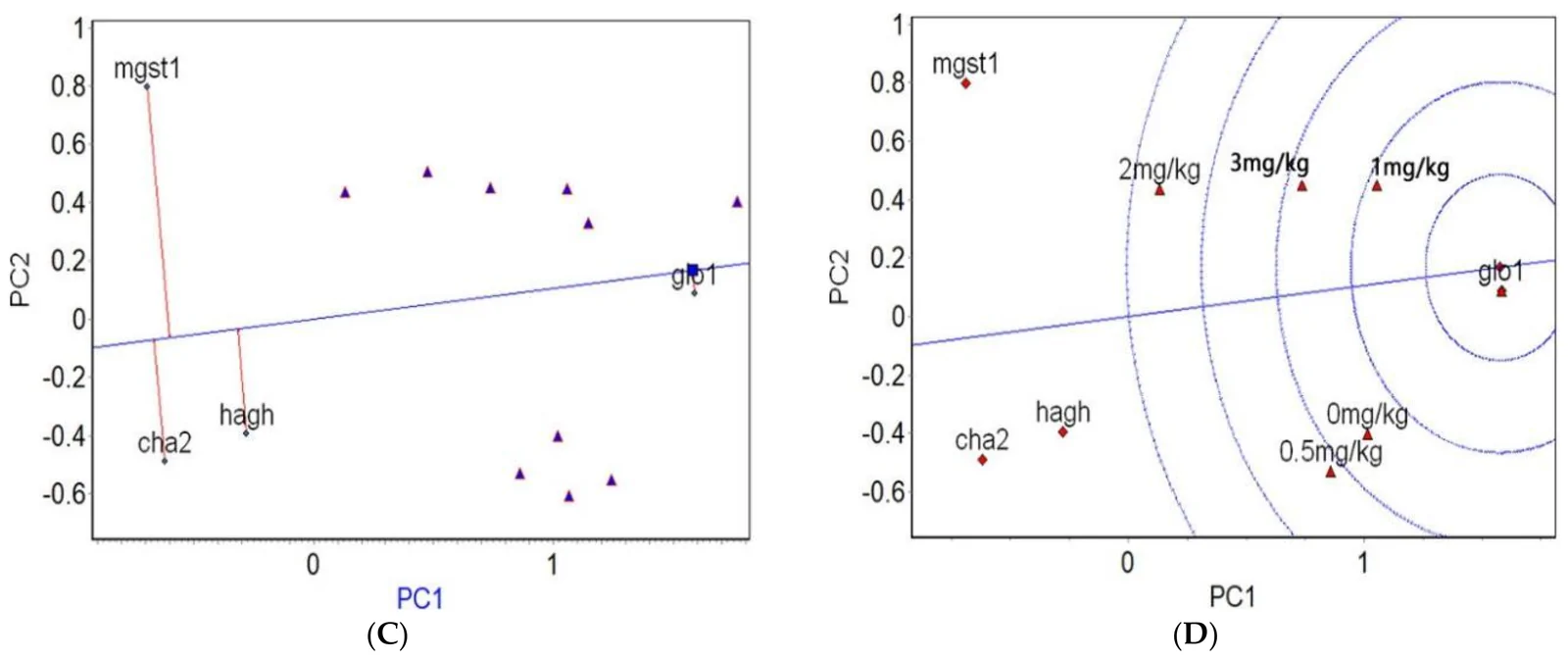
GGE biplot of selenium-related genes *mgst1*, *cha2*, *hagh*, and *glo1* in different selenium concentrations at 20 d. PC1 and PC2 explained 67.8% and 20.3% of the total variation, respectively. For detailed explanations of each view, see Supplementary Materials Section S2. (**A**) The relationship among different concentrations; (**B**) “Which-won-where” view; (**C**) “High expression and expression stability” view; (**D**) GGE biplot with concentric circles.

**Figure 5:**
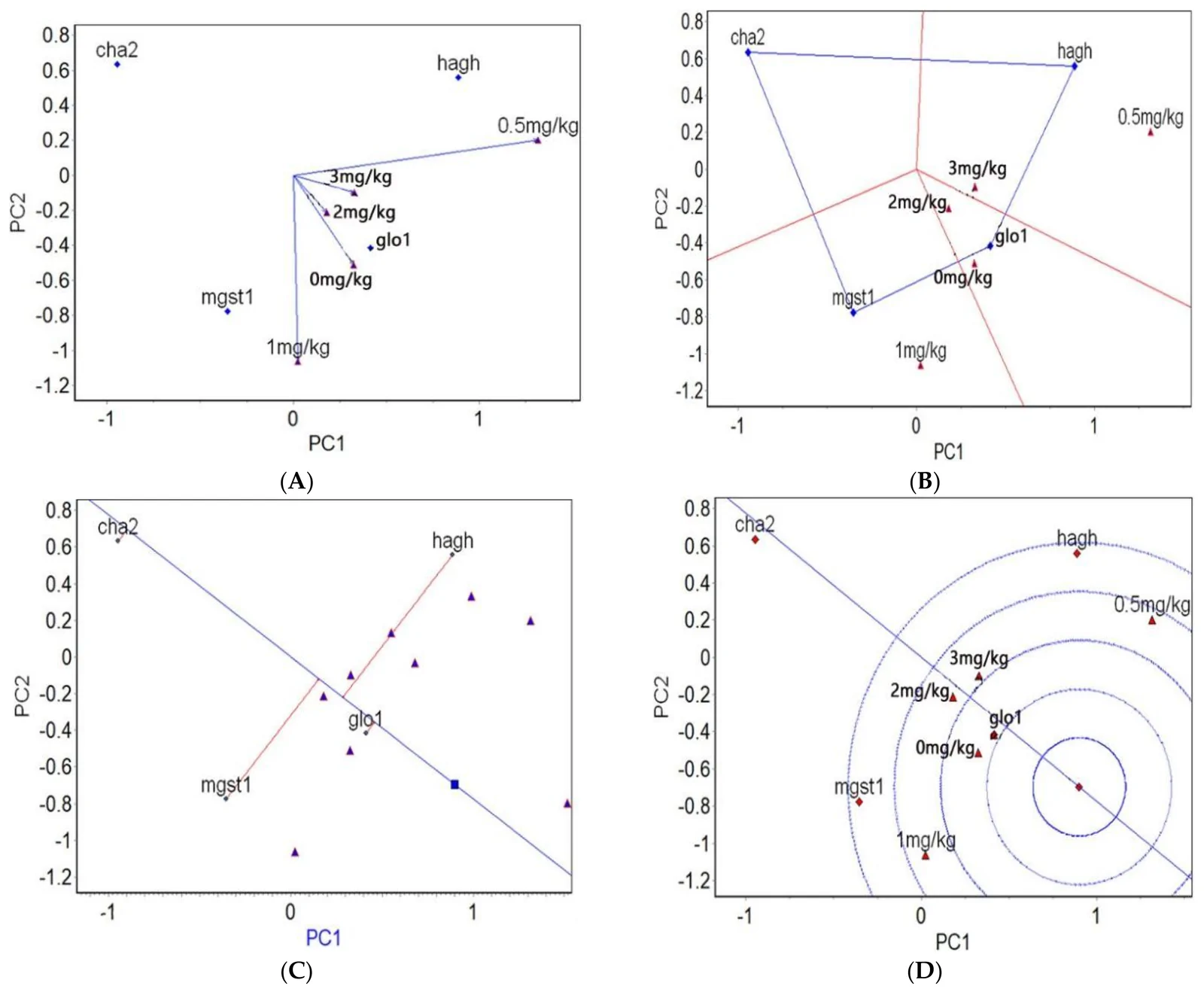
GGE biplot of selenium-related genes *mgst1*, *cha2*, *hagh*, and *glo1* in different selenium concentrations at 40 d. PC1 and PC2 explained 48.93% and 36.79% of the total variation, respectively. For detailed explanations of each view, see Supplementary Materials Section S2. (**A**) The relationship among different concentrations; (**B**) “Which-won-where” view; (**C**) “High expression and expression stability” view; (**D**) GGE biplot with concentric circles.

**Figure 6:**
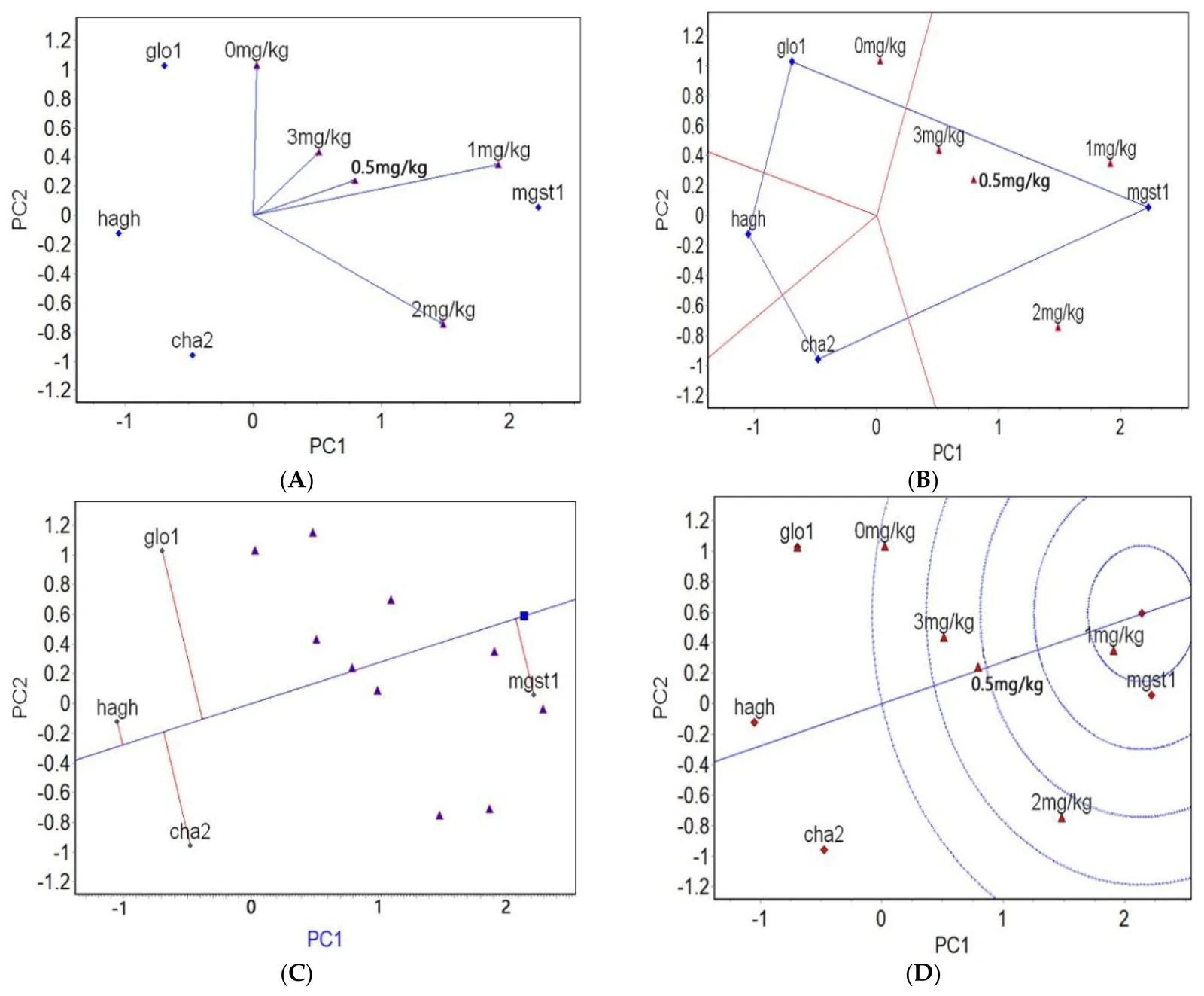
GGE biplot of selenium-related genes *mgst1*, *cha2*, *hagh*, and *glo1* in different concentrations at 60 d. PC1 and PC2 explained 69.08% and 20.39% of the total variation, respectively. For detailed explanations of each view, see Supplementary Materials Section S2. (**A**) The relationship among different concentrations; (**B**) “Which-won-where” view; (**C**) “High expression and expression stability” view; (**D**) GGE biplot with concentric circles.

GGE biplot analysis was carried out based on mean values of the four genes for the five selenium concentrations at different durations. The “relationship among different selenium concentrations,” “which-won-where,” “high expression and expression stability,” and “concentric circles” views of the GGE biplot (Figure 1, Figure 2, Figure 3, Figure 4, Figure 5 and Figure 6) were drawn based on the results of the GGE biplot analysis (Supplementary Tables S3 and S4). See Supplementary Materials Section S2 for a detailed explanation of the GGE biplot views.

At 20 d the “relationship among different selenium concentrations” view (Figure 1A) revealed that the angle between 0.5 mg/kg and 1 mg/kg was the smallest, indicating a basically similar ranking of the four genes expression in the two selenium concentrations. The “which-won-where” view (Figure 1B) revealed that the five selenium concentrations were divided into two regions: 0 mg/kg, 0.5 mg/kg, and 1 mg/kg in one group, and 2 mg/kg and 3 mg/kg in the other. The expression of *gpx1* and *selenop* is the highest in the 0 mg/kg–0.5 mg/kg–1 mg/kg region and 2 mg/kg–3 mg/kg region, respectively. The “high-expression and expression stability” view (Figure 1C) revealed that the expression of *gpx1* is the highest, followed by *selenop* and *trnau1apl*, and the expression of *selenoo1* is the most stable, followed by *gpx1* and *trnau1apl*. The “concentric circles” view (Figure 1D) revealed that *gpx1* had the strongest expression and stability, followed by *selenop* and *trnau1apl*.

At 40 d, the “relationship among different selenium concentrations” view (Figure 2A) revealed that the angles between 0 mg/kg and 0.5 mg/kg as well as 3 mg/kg and 0.5 mg/kg were relatively small, indicating the rankings of the expression of the four genes under these two pairs of selenium concentrations are relatively similar. The “which-won-where” view (Figure 2B) revealed that the five selenium concentrations were divided into two regions, with 2 mg/kg in one and the others in the other. The expression of gpx1 and selenop is the highest in the 2 mg/kg region and the 0–0.5–1–3 mg/kg region, respectively. The “high-expression and expression stability” view (Figure 2C) revealed that the expression of *selenop* is the highest, followed by *GPX1* and *trnau1apl*, and the expression of *selenoo1* is the most stable, followed by *trnau1apl* and *selenop*. The “concentric circles” view (Figure 2D) revealed that *selenop* had the strongest expression and stability, followed by *gpx1* and *trnau1apl*.

At 60 d, the “relationship among different selenium concentrations” view (Figure 3A) revealed that the angles between 1 mg/kg and 2 mg/kg, 0 mg/kg and 3 mg/kg as well as 0.5 mg/kg and 3 mg/kg were relatively small, indicating the rankings of the expression of the four genes under these three pairs of selenium concentrations are relatively similar. The “which-won-where” view (Figure 3B) revealed that the five selenium concentrations were divided into three regions, 0.5 mg/kg belonging to one region, 1 mg/kg and 2 mg/kg belonging to one region, 0 mg/kg belonging to one region, and 3 mg/kg does not belong to any region. The expression of *GPX1*, *trnau1apl* and *selenop* is the highest in the 0.5 mg/kg region, 1–2 mg/kg region and 0–3 mg/kg region, respectively. The “high-expression and expression stability” view (Figure 3C) revealed that the expression of *GPX1* is the highest, followed by *trnau1apl* and *selenop*, and the expression of *GPX1* is the most stable, followed by *selenoo1* and *selenop*. The “concentric circles” view (Figure 3D) revealed that *gpx1* had the strongest expression and stability, followed by *trnau1apl* and *selenop*.

### 3.2. Glutathione Metabolism-Related Genes (mgst1, cha2, hagh, and glo1)

#### 3.2.1. AMMI Analysis of Glutathione Metabolism-Related Genes

The AMMI analysis revealed that the expressions of the selenium-related genes *mgst1*, *cha2*, *hagh*, and *glo1* were extremely significantly affected by genotype, selenium concentration, and genotype × selenium concentration interactions at three durations (Table 4). At 20 d, 52.28125%, 24.70139% and 17.34659% of the total SS (sum of squares) were attributable to the genotype, selenium concentration effect, and the genotype × selenium concentration interaction, respectively; at 40 d, the values were 30.36026%, 20.9675% and 41.14476%; and at 60 d, they were 48.28776%, 16.86181% and 30.71218%.

#### 3.2.2. GGE Biplot Analysis of Glutathione Metabolism-Related Genes

At 20 d, the “relationship among different selenium concentrations” view (Figure 4A) revealed that the angles between 1 mg/kg and 3 mg/kg, as well as 0 mg/kg and 0.5 mg/kg, were relatively small, indicating that the rankings of the expression of the four genes under these two pairs of selenium concentrations are relatively similar. The “which-won-where” view (Figure 4B) revealed that the five selenium concentrations were divided into one region, 0 mg/kg, 0.5 mg/kg, 1 mg/kg and 3 mg/kg belonging to one region, and 2 mg/kg does not belong to any region. The expression of *glo1* is the highest in the region. The “high-expression and expression stability” view (Figure 4C) revealed that the expression of *glo1* is the highest, followed by *hagh* and *mgst1*, and the expression of *glo1* is the most stable, followed by *hagh* and *cha2*. The “concentric circles” view (Figure 4D) revealed that *glo1* had the strongest expression and stability, followed by *hagh* and *cha2*.

At 40 d, the “relationship among different selenium concentrations” view (Figure 5A) revealed that the angles between 0 mg/kg and 2 mg/kg was the smallest, indicating that the rankings of the expression of the four genes under the two selenium concentrations are very similar. The “which-won-where” view (Figure 5B) revealed that the five selenium concentrations were divided into three regions, 1 mg/kg belonging to one region, 0 mg/kg and 2 mg/kg belonging to one region, and 0.5 mg/kg and 3 mg/kg belonging to one region. The expression of *mgst1*, *glo1* and *hagh* is the highest in the 1 mg/kg region, 0 mg/kg–2 mg/kg region and 0.5–3 mg/kg region, respectively. The “high-expression and expression stability” view (Figure 5C) revealed that the expression of *glo1* is the highest, followed by *hagh* and *mgst1*, and the expression of *glo1* is the most stable, followed by *cha2* and *mgst1*. The “concentric circles” view (Figure 5D) revealed that *glo1* had the strongest expression and stability, followed by *hagh* and *mgst1*.

At 60 d, the “relationship among different selenium concentrations” view (Figure 6A) revealed that the angles between 0.5 mg/kg and 1 mg/kg was the smallest, indicating the rankings of the expression of the four genes under the two selenium concentrations are very similar. The “which-won-where” view (Figure 6B) revealed that the five selenium concentrations were divided into two regions, with 0 mg/kg constituting one region and the other selenium concentrations constituting the other. *glo1* expression is highest in the 0 mg/kg region, whereas *mgst1* expression is highest in the 0.5–1–2–3 mg/kg region. The “high-expression and expression stability” view (Figure 6C) revealed that the expression of *mgst1* is the highest, followed by *glo1* and *cha2*, and the expression of *hagh* is the most stable, followed by *mgst1* and *cha2*. The “concentric circles” view (Figure 6D) revealed that *mgst1* had the strongest expression and stability, followed by *glo1* and *cha2*.

## 4. Discussion

To elucidate the molecular mechanism by which nano-selenium regulates selenium metabolism in *P. dabryanus*, this study classified candidate genes into two categories. The first category comprises selenoprotein and synthesis-related genes (*selenoo1*, *trnau1apl*, *selenop*, *gpx1*), which specifically reflect the response of the selenoprotein pathway. The second category comprises glutathione metabolism-related genes (*mgst1*, *cha2*, *hagh*, *glo1*), which characterise the non-selenium-dependent antioxidant and detoxification systems.

In this paper, using AMMI and GGE biplot analysis, we examined the interactions between these two kinds of genes and selenium concentration, thereby distinguishing the regulatory effect of nano-selenium on selenoprotein-specific pathways and its inductive effect on glutathione metabolic pathways. This reveals the synergistic or compensatory relationship between the two genes in regulating selenium homeostasis in aquatic animals, providing a molecular basis for elucidating the underlying mechanisms by which nano-selenium affects selenium metabolism networks.

**Figure 7:**
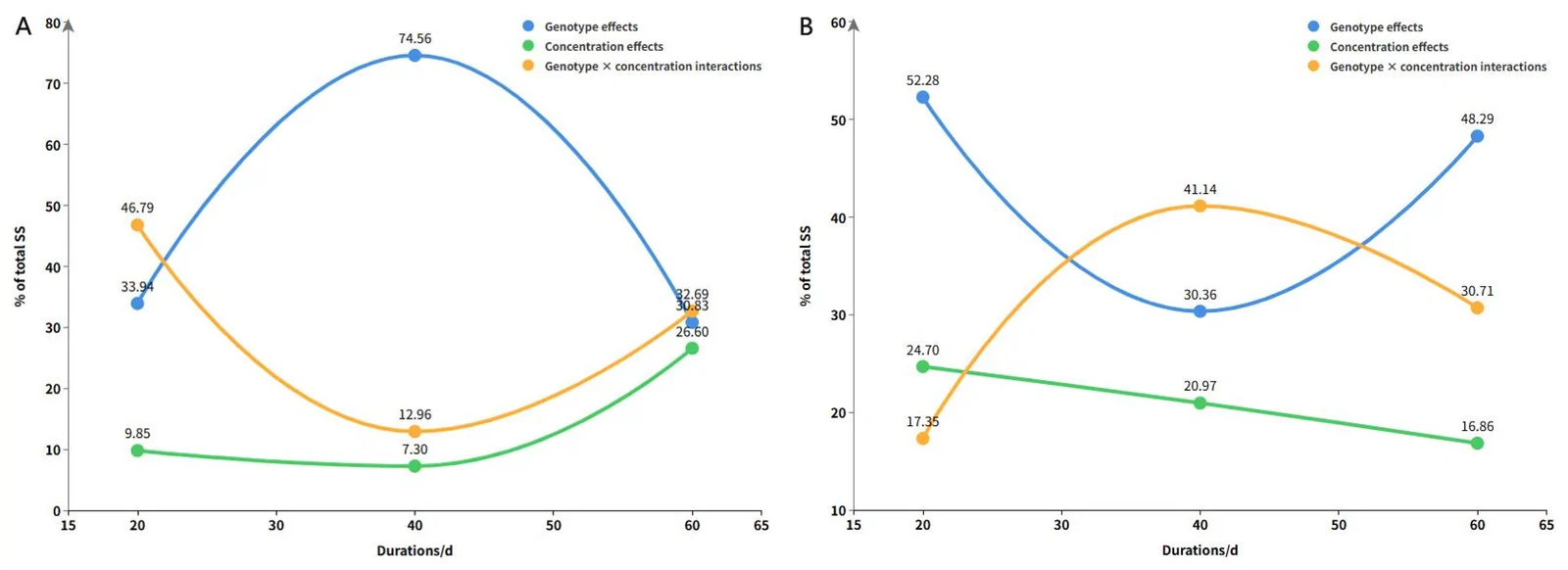
The genotype effect, selenium concentration effect, and genotype × selenium concentration interaction change with durations. Notes: (**A**) *selenoo1*, *trnau1apl*, *selenop*, and *gpx1* genes; (**B**) *mgst1*, *cha2*, *hagh*, and *glo1* genes.

The AMMI analysis of the expression of the two types of selenium-related genes in *P. dabryanus* showed that the three effects influenced gene expression levels differently between the two gene types (Figure 7). For selenoprotein-synthesis-related genes, the effect of selenium concentration shows a slight decrease followed by a gradual increase over time (Figure 7A). For glutathione metabolism-related genes, the effect of selenium concentration shows a linear decrease over time (Figure 7B). In both types of genes, as time progresses, there is a clear antagonistic relationship between the genotype effect and the genotype × selenium concentration interaction (i.e., their trends are opposite). However, the temporal trends of these two effects are completely opposite between the two gene types (Figure 7A,B). For selenoprotein-synthesis-related genes, the genotype effect initially increases and then decreases over the three time points, while the genotype × selenium concentration interaction initially decreases and then increases (Figure 7A). For glutathione metabolism-related genes, the opposite pattern is observed: the genotype effect first decreases and then increases, whereas the genotype × selenium concentration interaction first increases and then decreases (Figure 7B). On the whole, the temporal trends of the selenium concentration effect, the genotype effect, and their interaction are opposite between the two types of genes.

These contrasting temporal patterns represent the key empirical findings of this study. In the following discussion, we offer several possible explanations for these observed trends; however, these interpretations are speculative and based on previously reported mechanisms in the literature. One possible explanation is that the functional roles of the two gene categories in the selenium metabolic network differ. Selenoprotein-synthesis-related genes directly depend on selenocysteine (Sec) incorporation and selenium supply; we hypothesise that their expression may be limited by the efficiency of selenium conversion and translation, which might cause a “lag” in their response to selenium concentration and result in a relatively low overall effect. Studies in zebrafish have shown that the mRNA expression of gpx1 paralogues and GPX activity respond in a dose-dependent manner to dietary Se status [29], and selenoprotein expression can be influenced by Se bioavailability and metabolic efficiency [22]. The initial increase followed by a decrease in genotypic differences over time might reflect differential sensitivity to selenium utilisation among genetic backgrounds during the middle stage (40 days). In contrast, glutathione-metabolism-related genes are often rapid responders to oxidative stress, and we speculate that their expression is driven primarily by reactive oxygen species (ROS) levels. They might be rapidly induced during the early phase of nano-selenium exposure (20 days). As the selenoprotein system (especially gpx1) gradually exerts its antioxidant function and may scavenge ROS, the demand for these glutathione-related genes could subsequently decrease. This could explain why the effect of selenium concentration linearly weakened over time, and why genotypic differences were larger in the early and late stages but smallest in the middle stage. Taken together, the functional divergence and potential compensatory relationship between the two gene categories may underlie the opposite temporal trends observed. These mechanistic interpretations remain hypotheses that will require direct validation in future studies through measurements of ROS, GSH levels, and enzyme activities.

**Figure 8:**
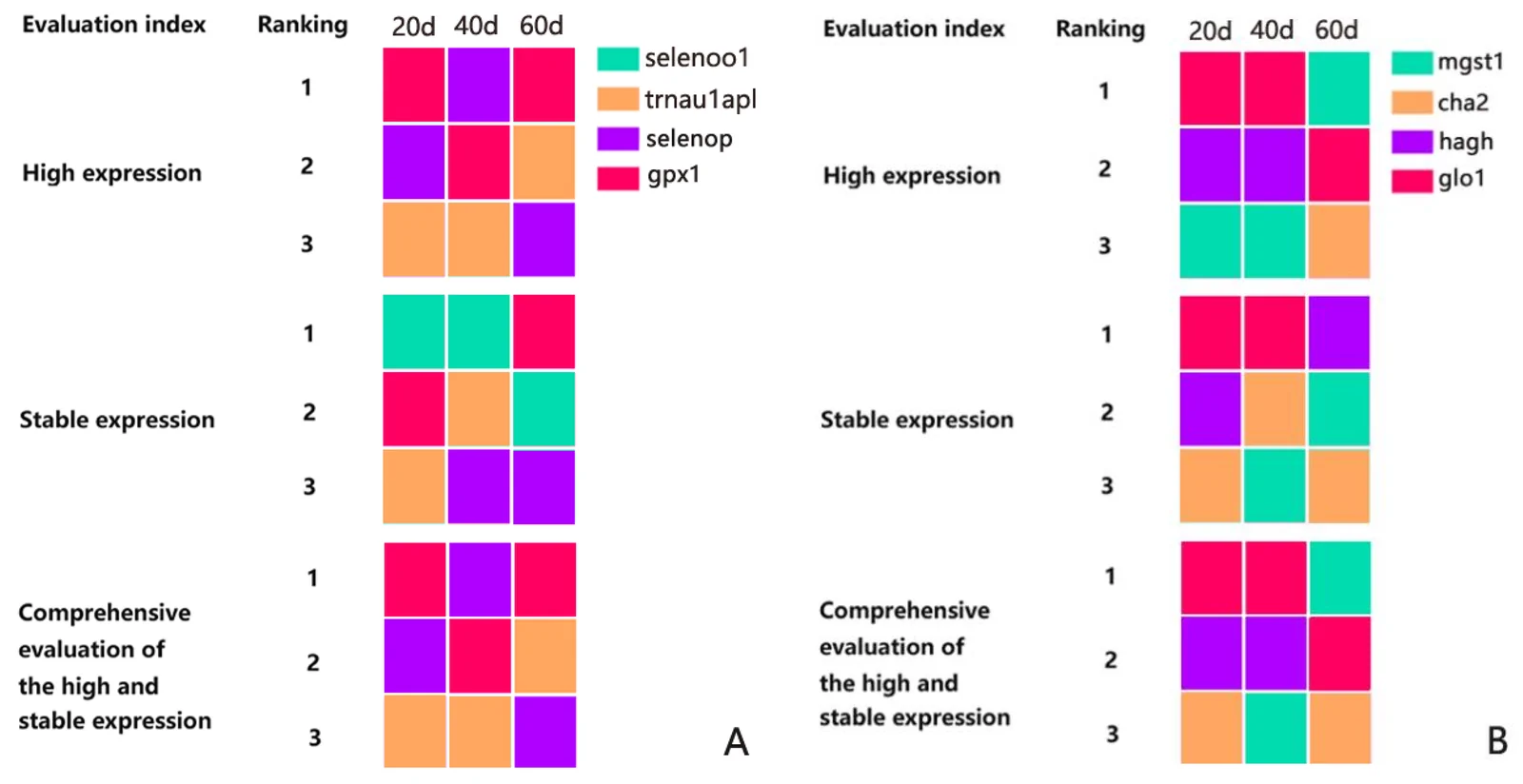
Rankings of stable-expression, high-expression, and the comprehensive evaluation of stable- and high-expression of the selenium-related genes at different durations. Notes: (**A**) *selenoo1*, *trnau1apl*, *selenop*, and *gpx1* genes; (**B**) *mgst1*, *cha2*, *hagh*, and *glo1* genes.

GGE biplot analysis indicated that the expression rankings of different selenium-related genes varied greatly across different selenium concentration gradients, regardless of whether they were involved in selenoprotein synthesis or glutathione metabolism (Figure 1, Figure 2, Figure 3, Figure 4, Figure 5 and Figure 6A). This indicates that distinct selenium-related genes respond differently to varying selenium concentrations, which is consistent with the diversity of selenoprotein gene expression patterns reported in other fish species [22,30]. Considering three time points, the two genes with the highest expression levels among selenoprotein-synthesis-related genes are *gpx1* and *selenop* (Figure 8A). This observation is consistent with studies in zebrafish, where dietary Se status significantly influences *gpx1* and *sepp1* expression, with *gpx1* responding in a dose-sensitive manner and *sepp1* showing paralogue-specific regulation [29]. We speculate that this may be because *gpx1* is the core selenium-containing enzyme that alleviates intestinal oxidative stress; exogenous selenium supplementation may rapidly upregulate *gpx1* to meet antioxidant demands in the foregut. Meanwhile, as a selenium transporter, selenop needs to be expressed in large amounts under selenium supplementation to distribute selenium to peripheral tissues. Both functions are important and selenium-sensitive, potentially explaining their high expression levels. Indeed, a promoter analysis study in yellow catfish demonstrated that Se directly increases selenop promoter activity through transcription factors including Nrf2 and FoxO4 [16]; this regulatory link further supports the sensitivity of selenop to dietary Se levels. The top two genes in terms of stable expression are *selenoo1* and *gpx1* (Figure 8A). Selenoo1 is an ER-resident selenoprotein primarily involved in basic protein folding and redox regulation within the endoplasmic reticulum [13]. Its constitutive-like expression pattern—less affected by fluctuations in exogenous selenium levels—is consistent with its role in maintaining ER homeostasis rather than responding to changes in selenium supply [31]. For *gpx1*, it is possible that its expression rapidly reaches a plateau within the experimental selenium range (0.5–3.0 mg/kg), so further dose increases do not significantly alter its expression level, resulting in minimal intergroup variability. A similar dose-saturation response for *gpx1* expression has been reported in zebrafish, where GPX activity and mRNA levels were lowest when dietary Se levels resulted in maximum growth, with a proposed bimodal response pattern [29]. The top two in terms of comprehensive evaluation of stable and high expression are *gpx1* and *selenop* (Figure 8A). Based on these expression results, among glutathione-metabolism-related genes, *glo1* may serve as a potential biomarker for responses to nano-selenium-induced oxidative stress or glutathione metabolic load, but this proposal requires confirmation by functional assays. This aligns with the emerging recognition in aquaculture nutrition that specific selenoprotein genes, particularly *gpx1*, can serve as sensitive molecular indicators of Se status [7]. Based on three time points, the top two glutathione metabolism-related genes with high expression levels are *glo1* and *hagh* (Figure 8B); *glo1* and *hagh* encode glyoxalase I and glyoxalase II, respectively, which together constitute the glyoxalase pathway that detoxifies methylglyoxal (MGO) in a glutathione-dependent manner, thereby regenerating GSH and protecting cells from reactive carbonyl stress [32]. It is possible as a hypothesis that nano-selenium supplementation increases tissue GSH levels, thereby providing substrates and cofactors that could drive high expression of both genes. Studies in plants have shown that Se pretreatment upregulates the methylglyoxal detoxification system by enhancing GSH levels and the activities of glyoxalase I and II [33,34]. By extension, we speculate that a similar selenium-induced enhancement of the glyoxalase pathway may occur in fish. In contrast, *mgst1* mainly responds to exogenous toxins, and *cha2* is reportedly activated only under severe GSH depletion; therefore, their induction might be weaker under the selenium supplementation conditions used here. The top two genes in terms of expression stability were *glo1* and *hagh* (Figure 8B). The underlying mechanism for this stability remains unclear. One untested hypothesis is that their expression may be modulated by the Nrf2/ARE pathway, as both genes contain putative antioxidant response elements [32], and nano-selenium is known to activate Nrf2 signalling. However, direct experimental evidence (e.g., promoter analysis, Nrf2 knockdown) is required to support this speculation. Alternatively, their stable expression could simply reflect a ceiling effect after rapid initial induction, independent of specific regulatory pathways. The top two genes in terms of comprehensive evaluation of stable and high expression are *glo1* and *hagh* (Figure 8B). We suggest that this is because both genes maintained high and uniform expression throughout the entire experimental period (20, 40, 60 days), whereas other genes showed low or fluctuating expression at certain time points and were consequently penalised by the comprehensive evaluation model. Based on these expression results, among glutathione-metabolism-related genes, *glo1* may serve as a potential biomarker for responses to nano-selenium-induced oxidative stress or glutathione metabolic load, but this proposal requires confirmation by functional assays (e.g., enzyme activity, GSH measurement). Recent reviews support the use of *glo1* as a functional biomarker for cellular glutathione metabolic load and methylglyoxal stress in the context of oxidative stress and redox signalling [32], and the application of gene expression-based biomarkers in nano-selenium supplemented aquafeeds is emerging as a powerful approach for monitoring fish nutritional status and health [7].

Overall, while our AMMI and GGE analyses provide novel insights into genotype-by-selenium-concentration interactions, the functional interpretations presented here should be considered hypothesis-generating. Direct measurements of tissue selenium, glutathione, and antioxidant enzyme activities will be essential to confirm the proposed roles of gpx1 and glo1 as biomarkers, and to establish whether the observed expression patterns translate into functional effects at the protein and metabolic levels.

## 5. Conclusions

(1) The AMMI analysis for the two types of selenium-related genes in *P. dabryanus* showed that, for selenoprotein-synthesis-related genes, the effect of selenium concentration decreased slightly and then gradually increased over time. For glutathione metabolism-related genes, the effect of selenium concentration decreased linearly over time. In both types of genes, as time progressed, there was a clear antagonistic relationship between the genotype effect and the genotype × selenium concentration interaction. However, the temporal trends of these two effects were completely opposite between the two gene types. For selenoprotein-synthesis-related genes, the genotype effect initially increased and then decreased over the three time points, while the genotype × selenium concentration interaction initially decreased and then increased. For glutathione metabolism-related genes, the opposite pattern was observed. Overall, the temporal trends of the selenium concentration effect, the genotype effect, and their interaction were opposite between the two types of genes. These opposite temporal patterns suggest a potential functional divergence or compensatory relationship between selenoprotein synthesis and glutathione metabolism pathways in response to dietary selenium.
(2) GGE biplot analysis indicated that the expression rankings of different selenium-related genes varied greatly across selenium concentration gradients, regardless of whether they were involved in selenoprotein synthesis or glutathione metabolism. Considering the three time points, the two genes with the highest expression levels among selenoprotein-synthesis-related genes were *gpx1* and *selenop*. The top two genes in terms of expression stability were *selenoo1* and *gpx1*, and the top two based on comprehensive evaluation of both stability and high expression were *gpx1* and *selenop*. Based on these results, among genes related to selenoprotein synthesis, *gpx1* may be a promising functional biomarker for nano-selenium bioavailability, selenium nutritional status, and antioxidant responses. For the three time points, the top two glutathione metabolism-related genes in terms of high expression levels, expression stability, and comprehensive evaluation were all *glo1* and *hagh*. Based on these results, among genes related to glutathione metabolism, glo1 may be a potential indicator of responses to nano-selenium-induced oxidative stress or glutathione metabolic load, but this requires confirmation through biochemical assays. We note that these conclusions are drawn from mRNA expression data and await biochemical and physiological validation (e.g., measurements of tissue selenium, GSH, GPX activity, and oxidative markers) to confirm the functional relevance of the proposed candidate biomarkers.

## Figures and Tables

**Table 1:** Experimental basic feed formula and nutritional components.

Ingredients (%)	A	B	C	D	E
fish meal	20.00	20.00	20.00	20.00	20.00
soybean meal	18.00	18.00	18.00	18.00	18.00
vegetable meal	10.00	10.00	10.00	10.00	10.00
chicken meal	5.00	5.00	5.00	5.00	5.00
fermented soybean meal	10.00	10.00	10.00	10.00	10.00
corn protein flour	10.00	10.00	10.00	10.00	10.00
bean oil	4.00	4.00	4.00	4.00	4.00
corn starch	16.00	16.00	16.00	16.00	16.00
yeast hydrolysate	2.00	2.00	2.00	2.00	2.00
CaH_2_PO_4_	2.00	2.00	2.00	2.00	2.00
choline chloride	0.50	0.50	0.50	0.50	0.50
vitamin premix and mineral premix *	1.00	1.00	1.00	1.00	1.00
sodium alginate	1.50	1.50	1.50	1.50	1.50
total	100.00	100.00	100.00	100.00	100.00
added Nano-Se (mg/kg)	0.00	0.50	1.00	2.00	3.00
measured total selenium content (mg/kg)	1.42	1.9	2.35	3.31	4.27

* The vitamin premix and mineral premix were added at 1% of the diet (10 g/kg). Therefore, the final concentration of each component in the complete diet is 1% of the values listed below. The premix provides per kg of premix: VA 16 000 IU, VD 33 000 IU, VC 200 mg, VE 200 mg, VK3 20 mg, VB1 13.4 mg, VB2 20 mg, VB6 30 mg, VB12 27 mg, D-calcium pantothenate acid 80 mg, nicotinic acid 130 mg, folic acid 6.6 mg, biotin 1000 mg, Inositol 270 mg, calcium lactate 1000 mg, Fe (as ferric citrate) 100 mg, Mg (as magnesium sulfate) 400 mg, Al (as aluminium chloride) 20 mg, Zn (as zinc chloride) 60 mg, Cu (as copper sulfate) 30 mg, Mn (as manganese sulfate) 20 mg, and Potassium iodide 20 mg.

**Table 2:** Primers for the eight selenium-related genes in the quantitative PCR.

Serial Number	Genes	Primer Sequence	TM (°C)
1	*selenoo1*	F: AGACAAGAGCACGTTGGTCC	60.25
		R: GCCAGCCGCTGTCTGTATTT	61.03
2	*trnau1apl*	F: CCTCCTCCAGTTCCACAAGG	59.67
		R: TTTGGATCTTCGGCAGGGTC	60.04
3	*selenop*	F: TTGACCTTCCATATTGTACTGCC	58.8
		R: TGCCGACTATTGTCTCCTGTG	58.5
4	*gpx1*	F:ACACCCGCTTTGGGAATCAA	60.1
		R:CGGACATATTTCAGAGAAAGGAGG	60
5	*mgst1*	F: CACGGTGGCTTACCTGATGT	60.04
		R: GGCCATGGAGAAGGTTGTGA	59.96
6	*cha2*	F: AATTACCTTGGCCCAGCTCC	60.03
		R: ATTTTTGCCACTGGGGCCTA	59.88
7	*hagh*	F: CCCAGAAGGTTGTGGATGCT	59.96
		R: AGTGATGGTGCGTGGTAAGG	60.04
8	*glo1*	F: CAATGGCAACTCAGATCCACG	61.2
		R: GGTCACTCCCTGCTCCTCAAA	61.4
9	*ef1α*	F: ACAGCAAGAACGACCCACC	60
		R: AAAGCGACCAAGAGGAGGAT	60
10	*β-actin*	F:TCTTGGGTATGGAGTCTTGCGGT	58
		R:TCTTGATTTTCATTGTGCTGGGG	58

## Data Availability

The raw data supporting the conclusions of this article will be made available by the authors on request.

## Supplementary Materials

The following supporting information can be downloaded at: https://www.mdpi.com/article/10.3390/ani16182837/s1, Table S1: Sequences of primers used for qRT-PCR, amplification efficiency, slope, and R^2^. Table S2: Expressions of selenium-related genes selenoo1, trnau1apl, selenop, and gpx1 in different concentrations at different durations (Raw data). Table S3: GGE biplot analysis of selenium-related genes *selenoo1*, *trnau1apl*, *selenop*, and *gpx1* in different concentrations at different durations. Table S4: GGE biplot analysis of selenium-related genes *mgst1*, *cha2*, *hagh*, and *glo1* in different concentrations at different durations.
