# Supplementary material for: Genotype by Selenium Concentration Interactions for Selenium-Related Genes in Paramisgurnus dabryanus at Different Durations
Source: Animals (Basel). 2026 Sep 9;16(18):2837. doi: 10.3390/ani16182837 (PMC13603655; doi:10.3390/ani16182837)
Supplement: Supplementary file 1 [file animals-16-02837-s001.zip › animals-4525816-supplementary.pdf]

Section S1

Table S1 Sequences of primers used for qRT-PCR, amplification efficiency, slope, and R^2

Genes	Amplification efficiency (%)	slopes	R^2
selenoo1	93.17	-3.49	0.97
trnau1apl	97.63	-3.38	0.97
selenop	95.67	-3.43	0.98
gpx1	107.28	-3.15	0.98
cha2	95.33	-3.43	0.97
hagh	100.5	-3.31	0.99
glo1	105.81	-3.19	0.99
mgst1	97.71	-3.37	0.99
ef1α	102.65	-3.26	0.97
β-actin	100.66	-3.31	0.97

Table S2. Expressions of selenium-related genes selenoo1, trnau1apl, selenop, and gpx1 in different concentrations at different durations (Raw data)

Durations	concentration	Gene	Repeat group 1	Repeat group 2	Repeat group 3	Repeat group 4
	0mg/kg	*selenoo1*	0.57965261	0.42114689	0.51424523	0.54191539
	0mg/kg	*trnau1apl*	1.77697172	0.9369141	1.23019556	1.11825841
	0mg/kg	*selenop*	0.86129737	1.33291366	1.35308268	0.77266592
	0mg/kg	*gpx1*	1.74344934	1.64274792	1.74344934	1.85032382
	0.5mg/kg	*selenoo1*	0.83546791	1.10356357	0.96020412	0.96020412
	0.5mg/kg	*trnau1apl*	1.00398958	1.21037402	0.98933003	0.79684669
	0.5mg/kg	*selenop*	0.60064453	0.64673653	0.64673653	0.69636552
	0.5mg/kg	*gpx1*	1.43671886	1.28887389	1.69283862	1.82274294
	1mg/kg	*selenoo1*	1.10273712	1.36693324	1.02603136	0.9561104
	1mg/kg	*trnau1apl*	2.99995312	2.91375277	2.20574283	1.22771472
20d	1mg/kg	*selenop*	0.98026927	0.98026927	0.89373354	1.07518382
	1mg/kg	*gpx1*	2.21657677	2.22855893	2.24405978	2.17765857
	2mg/kg	*selenoo1*	0.39985976	0.39985976	0.36786757	0.4346342
	2mg/kg	*trnau1apl*	0.27356635	0.24513103	0.3039625	0.27477028
	2mg/kg	*selenop*	1.86480888	2.39150145	1.88502458	1.43851806
	2mg/kg	*gpx1*	1.92675022	1.83542063	1.88053107	1.88053107
	3mg/kg	*selenoo1*	0.42158649	0.41002768	0.36244575	0.50420118
	3mg/kg	*trnau1apl*	0.55975616	0.51195224	0.56449608	0.42464746
	3mg/kg	*selenop*	3.67312134	2.71853094	2.83167982	3.04652374
	3mg/kg	*gpx1*	2.08999005	1.59954396	2.1637145	2.63776756
	0mg/kg	*selenoo1*	0.13747076	0.13451152	0.16584827	0.11645564
	0mg/kg	*trnau1apl*	0.68874865	0.3031577	0.42978076	0.44770517
	0mg/kg	*selenop*	1.36301889	1.74261033	1.17385157	1.23791878
	0mg/kg	*gpx1*	0.69884064	0.69884064	0.68541252	0.71253185
	0.5mg/kg	*selenoo1*	0.50721961	0.49837093	0.66743983	0.36563683
	0.5mg/kg	*trnau1apl*	0.5154017	0.47173068	0.41023813	0.70746897
	0.5mg/kg	*selenop*	1.62216061	1.85931153	1.82783886	2.16782756
	0.5mg/kg	*gpx1*	1.31556281	1.13179014	1.0171414	1.08343983
	1mg/kg	*selenoo1*	0.13556891	0.13225696	0.18029501	0.1044908
	1mg/kg	*trnau1apl*	0.40713763	0.27229722	0.33296012	0.33296012
40d	1mg/kg	*selenop*	1.2649039	1.0361623	1.51003641	1.29347305
	1mg/kg	*gpx1*	1.1259253	1.23351715	1.02771799	1.1259253
	2mg/kg	*selenoo1*	0.20603332	0.24419353	0.30554628	0.23130658
	2mg/kg	*trnau1apl*	0.23234725	0.45175036	0.42453912	0.39111416
	2mg/kg	*selenop*	1.80406335	1.78334192	1.8348911	1.92019124
	2mg/kg	*gpx1*	2.23888089	2.11322216	2.59567744	2.04595977
	3mg/kg	*selenoo1*	0.25123905	0.17897433	0.22773713	0.21715537
	3mg/kg	*trnau1apl*	0.26321451	0.30762295	0.32080783	0.28844817
	3mg/kg	*selenop*	2.14292793	1.54890644	2.14292793	2.96476276
	3mg/kg	*gpx1*	1.36867298	1.03009527	1.47711052	1.27701704
	0mg/kg	*selenoo1*	0.22046503	0.18095489	0.18395113	0.33026231
	0mg/kg	*trnau1apl*	0.32190353	0.35109058	0.21815097	0.29104983
	0mg/kg	*selenop*	1.23221158	1.00318198	1.11825561	1.04336938
	0mg/kg	*gpx1*	0.7860962	0.91347962	0.60267179	0.7564005

	0.5mg/kg	*selenoo1*	0.41001311	0.48486506	0.37125606	0.38291182
	0.5mg/kg	*trnau1apl*	0.8840638	0.82588329	0.73865761	0.55198495
	0.5mg/kg	*selenop*	0.67394111	0.65576463	0.69636552	0.67031758
	0.5mg/kg	*gpx1*	1.03657277	1.27467557	1.40676136	0.84294635
	1mg/kg	*selenoo1*	0.43333273	0.42579442	0.39912377	0.34458893
	1mg/kg	*trnau1apl*	2.63761758	2.37929693	2.63026768	1.94148552
60d	1mg/kg	*selenop*	0.99778823	1.00086681	1.00086681	0.99165945
	1mg/kg	*gpx1*	1.5368192	1.79363141	1.82375778	1.39887735
	2mg/kg	*selenoo1*	0.60383568	0.370539	0.64609497	0.91965732
	2mg/kg	*trnau1apl*	1.64365263	1.64365263	1.46434625	1.84491473
	2mg/kg	*selenop*	0.99395332	0.76910368	0.95787989	0.89787302
	2mg/kg	*gpx1*	1.60380372	2.33854841	1.08047171	1.21792324
	3mg/kg	*selenoo1*	0.40905904	0.67416149	0.60670769	0.55103156
	3mg/kg	*trnau1apl*	0.8142712	0.80703603	0.84491227	0.79177664
	3mg/kg	*selenop*	0.92346386	1.39403105	0.8457296	0.9521182
	3mg/kg	*gpx1*	0.86002758	1.26379979	0.88963189	0.76160498

Table S3. GGE biplot analysis of selenium-related genes *selenoo1*, *trnau1apl*, *selenop*, and *gpx1* in different concentrations at different durations.

Durations	genes/ concentration	Mean expression	Deviation	PCA1	PCA2	PCA3	Distance from center point (D_i)
	selenoo1	0.6834	-0.6169	-0.8006	-0.63827	0.364587	1.086864
	trnau1apl	1.0784	-0.2219	-0.81581	0.424734	-0.45514	1.026208
	selenop	1.5345	0.2341	1.056753	-0.57133	-0.26172	1.229489
	gpx1	1.9051	0.6048	0.55966	0.784867	0.352275	1.026321
20d	0mg/kg	1.1512	-0.1492	0.246196	0.62858	-0.10602	0.683349
	0.5mg/kg	1.0432	-0.2571	-0.01125	0.433641	0.519512	0.676804
	1mg/kg	1.6622	0.3618	-0.18955	0.95458	-0.25159	1.00521
	2mg/kg	1.1127	-0.1877	0.917468	0.16625	0.352515	0.996822
	3mg/kg	1.5325	0.2321	1.340911	-0.09058	-0.25294	1.367562
	Contribution rate (%)			**57.01**	**31.88**	**11.10**	
	selenoo1	0.2504	-0.6653	0.907053	0.068231	-0.30568	0.959605
	trnau1apl	0.4	-0.5157	0.735374	0.119257	0.334199	0.816508
	selenop	1.7121	0.7964	-1.05923	0.543939	-0.03125	1.191138
	gpx1	1.3002	0.3845	-0.5832	-0.73143	0.002734	0.935475
40d	0mg/kg	0.671	-0.2446	-0.49521	0.35808	0.35239	0.705434
	0.5mg/kg	1.0105	0.0949	-0.63485	0.332676	-0.171	0.73685
	1mg/kg	0.7197	-0.1959	-0.57938	-0.09599	0.161932	0.60919
	2mg/kg	1.1764	0.2608	-0.97539	-0.68792	-0.00654	1.193595
	3mg/kg	1.0005	0.0848	-0.94354	0.358313	-0.16257	1.022294
	Contribution rate (%)			**72.8**	**21.9**	**5.3**	
	selenoo1	0.4474	-0.4973	-0.88239	-0.47306	0.106596	1.006858
	trnau1apl	1.1813	0.2365	0.837834	-0.41617	-0.22389	0.96192
	selenop	0.9409	-0.0038	-0.33853	0.567328	-0.32802	0.737605
	gpx1	1.2094	0.2647	0.38309	0.321902	0.445309	0.669835
60d	0mg/kg	0.5971	-0.3477	-0.01927	0.776323	-0.16831	0.794592
	0.5mg/kg	0.7442	-0.2006	0.271727	0.294561	0.442697	0.597145
	1mg/kg	1.3585	0.4137	1.116376	-0.11399	-0.2497	1.149626
	2mg/kg	1.1873	0.2425	0.638816	0.034965	0.262608	0.691571
	3mg/kg	0.8368	-0.1079	0.115571	0.344683	-0.10846	0.379376
	Contribution rate (%)			**59.42**	**28.05**	**12.5**	

Table S4. GGE biplot analysis of selenium-related genes *mgst1*, *cha2*, *hagh*, and *glo1* in different concentrations at different durations.

Durations	genes/ concentration	Mean expression	Deviation	PCA1	PCA2	PCA3	Distance from center point (D_i)
20d	*mgst1*	0.9053	-0.461	-0.69029	0.79625	-0.00278	1.053811
	cha2	0.8446	-0.5217	-0.61904	-0.48916	-0.49756	0.932768
	hagh	1.1363	-0.23	-0.27734	-0.39382	0.592167	0.76333
	glo1	2.579	1.2127	1.586673	0.086727	-0.09183	1.591693
	0mg/kg	1.4052	0.0389	1.018282	-0.40133	-0.44113	1.180066
	0.5mg/kg	1.2155	-0.1508	0.860627	-0.52947	0.43614	1.100562
	1mg/kg	2.1738	0.8075	1.056364	0.44821	-0.20663	1.165974
	2mg/kg	0.6526	-0.7137	0.133659	0.435619	-0.09192	0.464841
	3mg/kg	1.3844	0.0181	0.736759	0.451494	0.413167	0.957793
	Contribution rate (%)			**67.8**	**20.3**	**11.9**	
40d	*mgst1*	1.0222	0.0701	-0.35449	-0.77508	0.403156	0.942839
	cha2	0.3398	-0.6123	-0.945	0.632703	-0.12792	1.144423
	hagh	1.123	0.1709	0.88656	0.558148	0.286456	1.086082
	glo1	1.3234	0.3713	0.412928	-0.41577	-0.56169	0.811705
	0mg/kg	1.0426	0.0905	0.326421	-0.5109	0.407135	0.730292
	0.5mg/kg	1.4751	0.523	1.314354	0.19969	0.060428	1.330809
	1mg/kg	0.9427	-0.0094	0.023292	-1.06285	-0.04556	1.064076
	2mg/kg	0.5874	-0.3647	0.181267	-0.21253	-0.5313	0.600252
	3mg/kg	0.7127	-0.2394	0.328192	-0.09877	-0.35026	0.490051
	Contribution rate (%)			**48.93**	**36.79**	**14.28**	
60d	*mgst1*	4.0186	2.1173	2.222115	0.055909	-0.13229	2.226752
	cha2	1.2036	-0.6977	-0.47741	-0.95761	0.513146	1.186698
	hagh	0.8684	-1.0328	-1.0502	-0.12604	-0.77156	1.309242
	glo1	1.5145	-0.3868	-0.69451	1.027739	0.390706	1.300476
	0mg/kg	0.9615	-0.9398	0.030146	1.030449	0.550464	1.16865
	0.5mg/kg	1.3088	-0.5925	0.797084	0.239887	0.028485	0.832887
	1mg/kg	2.9471	1.0458	1.911207	0.350028	-0.03055	1.943236
	2mg/kg	2.5008	0.5995	1.483208	-0.75032	0.289105	1.687148
	3mg/kg	1.7883	-0.113	0.511962	0.432905	-0.80027	1.044008
	Contribution rate (%)			**69.08**	**20.39**	**10.53**	

Section S2

The results of the GGE biplot analysis of the relationship among different concentrations (panel A, Figures 1–6) mainly reveal the similarity of gene expression among concentrations. The included angle of the two line segments indicates the correlation of the gene expression ranking in the concentration represented by the two line segments. When the included angle of the two segments is acute, the gene expression ranking in the two concentrations is positively correlated: the smaller the angle, the higher the correlation and the closer the gene expression ranking. When the angle between the two segments is obtuse, the gene expression ranking is negatively correlated in the two concentrations, and when the angle between the two segments is a right angle, the gene expression ranking in the two concentrations is not related. The which-won-where view of the GGE biplot (panel B, Figures 1–6) divides the experimental regions according to genotype × concentration interaction and identifies genes with the highest expression level in each region. The gene located at the top corner of the polygon in each region is the gene with the highest expression in this region.

The high expression and expression stability biplot (panel C, Figures 1–6) shows the genes with high and stable expression. The direction of the transverse oblique line to the ideal value is the approximate average expression of the gene in all concentrations: the closer to the ideal value, the higher the average gene expression. The straight line perpendicular to the transverse slash represents the tendency of the gene × concentration interaction, and a greater deviation of the vertical line from the transverse oblique line indicates greater instability of gene expression. The GGE biplot with concentric circles (panel D, Figures 1–6) comprehensively evaluates high expression and expression stability based on the distance of various genes from the central point of the genes. A smaller distance indicates higher and more stable gene expression.
